# First-line trifluridine/tipiracil + bevacizumab in patients with unresectable metastatic colorectal cancer: final survival analysis in the TASCO1 study

**DOI:** 10.1038/s41416-022-01737-2

**Published:** 2022-04-19

**Authors:** E. Van Cutsem, I. Danielewicz, M. P. Saunders, P. Pfeiffer, G. Argilés, C. Borg, R. Glynne-Jones, C. J. A. Punt, A. J. Van de Wouw, M. Fedyanin, D. Stroyakovskiy, H. Kroening, P. Garcia-Alfonso, H. Wasan, A. Falcone, R. Fougeray, A. Egorov, N. Amellal, V. Moiseyenko

**Affiliations:** 1 University Hospitals Leuven and KU Leuven, Leuven, Belgium; 2 Wojewodzkie Hospitals in Gdyni/Gdansk Medical University, Gdynia, Poland; 3 Christie Hospital NHS Foundation Trust, Manchester, UK; 4 Odense University Hospital, Odense, Denmark; 5 Vall d’Hebrón Institute of Oncology and Vall d’Hebrón University Hospital, Barcelona, Spain; 6 University Hospital Besançon, Besançon, France; 7 Mount Vernon Hospital, Northwood, UK; 8 Amsterdam University Medical Centres, Amsterdam, The Netherlands; 9 Julius Centre, University Medical Centre Utrecht, Utrecht University, Utrecht, The Netherlands; 10 VieCuri Medical Centre, Noord-Limburg, Venlo, The Netherlands; 11 NN Blokhin National Medical Research Centre of Oncology, Moscow, Russia; 12 Moscow City Oncology Hospital N62, Moscow, Russia; 13 Group Practice for Haematology and Oncology, Magdeburg, Germany; 14 Gregorio Marañón General University Hospital, Madrid, Spain; 15 Hammersmith Hospital, Imperial College London, London, UK; 16 Department of Oncology, University Hospital of Pisa, Pisa, Italy; 17 Servier International Research Institute, Suresnes, France; 18 Saint Petersburg Scientific Practical Centre for Specialized Medical Care, St Petersburg, Russia

**Keywords:** Colorectal cancer, Colorectal cancer

## Abstract

**Background:**

Therapeutic options are limited in patients with unresectable metastatic colorectal cancer (mCRC) ineligible for intensive chemotherapy. The use of trifluridine/tipiracil plus bevacizumab (TT-B) in this setting was evaluated in the TASCO1 trial; here, we present the final overall survival (OS) results.

**Methods:**

TASCO1 was an open-label, non-comparative phase II trial. Patients (*n* = 153) were randomised 1:1 to TT-B (trifluridine/tipiracil 35 mg/m^2^ orally twice daily on days 1–5 and 8–12, and bevacizumab intravenously 5 mg/kg on days 1 and 15 of each 28-day cycle) or capecitabine plus bevacizumab (C-B; capecitabine, 1250 mg/m^2^ orally twice daily on days 1–14 and bevacizumab 7.5 mg/kg intravenously on day 1 of each 21-day cycle). Final OS was analysed when all patients had either died or withdrawn from the study. Adjusted multivariate regression was used to investigate the effects of pre-specified variables on OS.

**Results:**

At 1 September 2020, median OS was 22.3 months (95% CI: 18.0–23.7) with TT-B and 17.7 months (95% CI: 12.6–19.8) with C-B (adjusted HR 0.78; 95% CI: 0.55–1.10). No variables negatively affected OS with TT-B. Safety results were consistent with prior findings.

**Conclusions:**

TT-B is a promising therapeutic regimen in mCRC patients ineligible for intensive chemotherapy.

**Clinical trial information:**

NCT02743221 (clinicaltrials.gov)

## Background

Standard first-line treatment for patients with unresectable metastatic colorectal cancer (mCRC) is a doublet or triplet chemotherapy regimen consisting of a fluoropyrimidine (capecitabine, or infusional 5-fluorouracil plus leucovorin) in combination with oxaliplatin and/or irinotecan [1–3]. Additionally, a biological agent targeted against vascular endothelial growth factor (VEGF; e.g. bevacizumab) or epidermal growth factor receptor (EGFR; e.g. cetuximab or panitumumab for the *RAS* wild-type subpopulation) should also be given, unless contraindicated. With such intensive first-line regimens, overall survival (OS) times of ~30 months have been achieved in clinical trials through the implementation of a treatment plan that typically consists of sequential lines of therapy tailored to patient and disease characteristics, as well as treatment history [1].

However, patients with untreated, unresectable mCRC are often considered ineligible for intensive chemotherapy because of their age or frailty, performance status, comorbidities, low unresectable tumour burden or other factors; additionally, some patients decline intensive chemotherapy because of concerns about toxicity. Some estimates suggest that, in practice, 50–60% of patients are not eligible for intensive treatment [4, 5]. Ineligible patients are often under-represented in clinical trials [6–11], and innovative, evidence-based treatment options in such patients are therefore more limited. In this setting, recommended first-line treatment often consists of fluoropyrimidine in combination with bevacizumab [1, 2]. In a recent meta-analysis, the median OS associated with such treatment was 20.4 months (95% confidence interval [CI]: 17.3–24.8 months) in patients ineligible for intensive chemotherapy because of age or frailty [9]. There is therefore a need to expand the range of treatment options for this patient population.

Trifluridine/tipiracil may be used in patients with mCRC who have been previously treated with, or are not considered candidates for, standard antineoplastic treatment options [12]. Unlike the mechanism of action of fluoropyrimidines, trifluridine undergoes intracellular, stepwise phosphorylation and subsequent incorporation into DNA [13], resulting in DNA dysfunction. Animal CRC xenograft studies have shown that intracellular concentrations of active (phosphorylated) trifluridine are augmented when it is co-administered with bevacizumab [14]; thus, there is a pharmacological rationale for combining trifluridine/tipiracil with bevacizumab in patients with mCRC.

Encouraging results in preclinical studies [14] and in patients with refractory mCRC [15] have led to the study of trifluridine/tipiracil plus bevacizumab (TT-B) as a first-line treatment in patients with mCRC who are ineligible for intensive chemotherapy. The ongoing phase III SOLSTICE trial (NCT03869892) is investigating the efficacy and safety of TT-B versus capecitabine plus bevacizumab (C-B) in this setting [16]. SOLSTICE follows on from the phase II TASCO1 (TAS-102 in COlorectal cancer 1) trial. The main findings of TASCO1 were reported in 2020: median (range) PFS was 9.2 (7.6–11.6) months in the TT-B group and 7.8 (5.5–10.1) months in the C-B group. Median (range) OS was 18 (15.2–NA) and 16.2 (12.5–NA) months, respectively [17]. The purpose of this article is to present the final OS analysis of the TASCO1 trial, performed after the last patient had withdrawn from the study.

## Methods

### Study design and participants

The design and methodology of the TASCO1 trial have been described in detail previously [17]. Briefly, this was a multinational, open-label, randomised, non-comparative phase II trial conducted at 52 participating centres in several European countries plus Australia and Brazil. The study was registered with ClinicalTrials.gov (registration number NCT02743221).

The study protocol was reviewed and approved by the Institutional Review Board at each participating centre, and was performed in accordance with the Declaration of Helsinki and Good Clinical Practice guidelines. All patients provided written informed consent to participate. An independent data safety monitoring board provided frequent oversight of the study.

Patients were eligible for inclusion if they were aged ≥18 years, had a recent diagnosis of unresectable mCRC, had known *RAS* status and had not received systemic anticancer therapy. Patients had to be deemed ineligible for standard first-line intensive chemotherapy containing irinotecan or oxaliplatin on the basis of age, low tumour burden, Eastern Cooperative Oncology Group (ECOG) performance status, comorbidities or other factors.

**Fig. 1:**
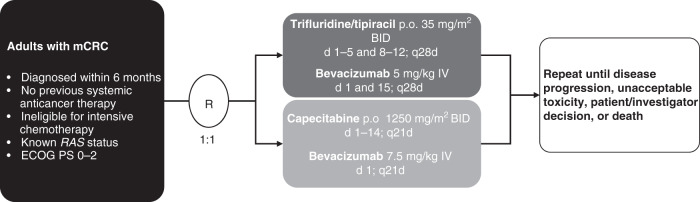
Design of the TASCO1 trial. BID twice daily, d days, ECOG Eastern Cooperative Oncology Group, IV intravenous, mCRC metastatic colorectal cancer, p.o. orally, PS performance status, qxd every x days, R randomisation.

Patients were randomised (1:1) using an interactive web response system to receive either TT-B or C-B, with cycles repeated every 4 weeks or 3 weeks, respectively, until disease progression, unacceptable toxicity, withdrawal, or death (Fig. 1). Dosages were as follows: trifluridine/tipiracil 35 mg/m^2^ orally twice daily on days 1–5 and 8–12 of each 28-day TT-B cycle; and capecitabine 1250 mg/m^2^ orally twice daily on days 1–14 of each 21-day C-B cycle (a starting dose for capecitabine of 1000 mg/m^2^ was allowed). Bevacizumab was administered intravenously at a dosage of 5 mg/kg every 2 weeks on days 1 and 15 of each cycle in the TT-B arm and at a dosage of 7.5 mg/kg on day 1 of each 21-day cycle in the C-B arm. Haematological support was provided according to general clinical practice. Patients were stratified at randomisation by *RAS* status (mutant vs wild-type), ECOG performance status (0, 1 or 2), and country.

The primary endpoint, progression-free survival (PFS), was analysed after 100 events (radiological progression or death) had occurred. The results of the primary analysis have been published, as have analyses of the secondary endpoints of overall response rate, disease control rate, duration of response, quality of life and safety [17]. OS, defined as the time in months from randomisation to death from any cause, was also a secondary endpoint. In the primary analysis, at the cut-off date of 20 January 2018, there were 22 deaths in the TT-B arm and 33 in the C-B arm (55 events in total); corresponding to an estimated median OS (95% CI) of 18.0 (15.2–not calculable) months and 16.2 (12.5–not calculable) months, respectively [17]. A final analysis of OS was performed at the end-of-study cut-off date of 1 September 2020 (i.e. once all participants had either died or withdrawn from the study) and forms the basis of this report.

An updated safety analysis was also performed on the final dataset. Treatment-emergent adverse events (TEAEs) were monitored throughout the study; severity (using National Cancer Institute Common Terminology Criteria for Adverse Events version 4.03 [18]) was assessed by study investigators.

### Statistical analysis

One hundred PFS events were required to describe the difference between the two arms; in order to observe this number of events, ~150 patients were randomised [17]. However, the trial was not designed to confirm a statistical difference between study treatments, and was therefore considered non-comparative. OS was analysed for all randomised patients who received at least one dose of study medication; the analysis was performed according to the originally assigned treatment arm. Hazard ratios (HRs) and corresponding 95% CIs were estimated using a Cox proportional hazards model adjusted according to *RAS* status and ECOG performance status. Kaplan–Meier estimates of OS were summarised for both treatment arms. An adjusted multivariate Cox regression analysis, based on a stepwise selection procedure, was used to investigate the effect of pre-specified factors on OS, including *RAS* status, ECOG performance status, sex, age group, geographical region and other baseline patient or disease characteristics. Statistical analyses were performed using SAS (version 9.2; SAS Institute, Cary, NC).

## Results

### Patient disposition and baseline characteristics

Between 29 April 2016 and 29 March 2017, a total of 153 patients were enrolled and randomised, 77 to the TT-B arm and 76 to the C-B arm. At the cut-off date of 1 September 2020, 132 patients (86.3%; *n* = 66 in each treatment arm) had died and 21 patients (13.7%; 11 in the TT-B arm and 10 in the C-B arm) were still alive and censored for the OS analysis. The median number of cycles was 8.0 in the TT-B arm and 9.0 in the C-B arm, and the median treatment duration was 34.6 weeks and 25.6 weeks, respectively.

Baseline characteristics of the study population have been described previously [17]. Briefly, patients had a median age of 75.0 years (range: 33.0–91.0 years) and more than half were male (*n* = 88; 57%) and white (*n* = 144; 97%). The majority had an ECOG performance status of 0 or 1 (*n* = 129; 84%), mutated *RAS* (*n* = 87; 57%) and ≥3 metastatic sites (*n* = 111; 73%). In the TT-B arm, the most common reasons for ineligibility for intensive treatment were older age (*n* = 28; 36%), low tumour burden (*n* = 15; 20%) and ECOG performance status (*n* = 14; 18%). In the C-B arm, more patients were ineligible because of their age (*n* = 42; 55%), but fewer were ineligible because of their performance status (*n* = 2; 3%) [17].

### Overall survival

**Fig. 2:**
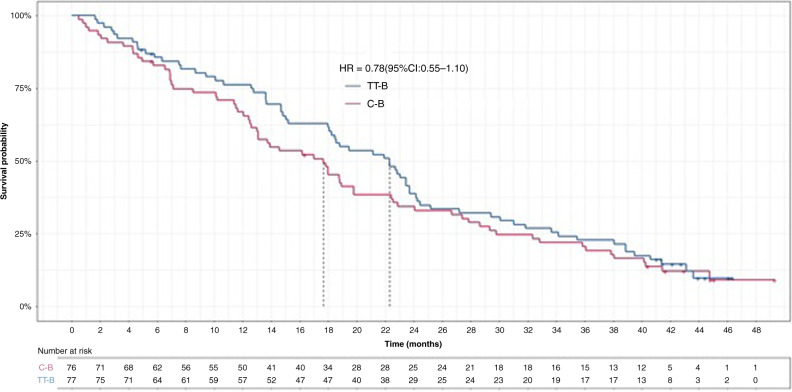
Kaplan–Meier estimates of overall survival probability (final analysis) for first-line trifluridine/tipiracil plus bevacizumab (TT-B; *n* = 77) and capecitabine plus bevacizumab (C-B; *n* = 76) in patients with metastatic colorectal cancer who were ineligible for intensive chemotherapy. CI confidence interval, HR hazard ratio.

Kaplan–Meier estimates of OS probability are shown in Fig. 2 and Table 1. Median OS was 22.3 (95% CI: 18.0–23.7) months in the TT-B arm, and 17.7 (95% CI: 12.6–19.8) months in the C-B arm (adjusted HR 0.78; 95% CI: 0.55–1.10). The delta for median OS (i.e. the difference between the TT-B and C-B arms) was 4.6 months. These results were consistent with estimated OS probabilities at 6, 12, 18 and 24 months (Table 1).

**Table 1:** Estimated overall survival probability at specified time-points (final analysis).

Time-point (months)	Overall survival probability (95% CI)	
	TT-B (*n* = 77)	C-B (*n* = 76)
6	0.86 (0.76–0.92)	0.83 (0.72–0.90)
12	0.76 (0.65–0.84)	0.67 (0.55–0.76)
18	0.63 (0.51–0.73)	0.47 (0.35–0.57)
24	0.39 (0.28–0.50)	0.34 (0.24–0.45)

*C-B* capecitabine plus bevacizumab, *CI* confidence interval, *TT-B* trifluridine/tipiracil plus bevacizumab.

**Fig. 3:**
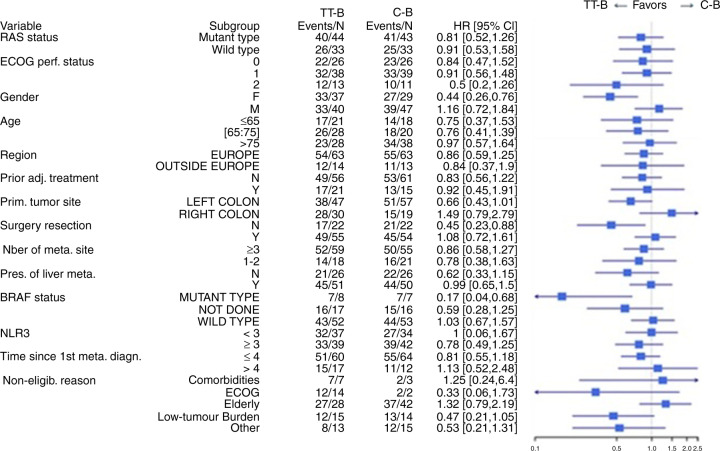
Forest plot of overall survival (OS) by subgroup. C-B capecitabine plus bevacizumab, CI confidence interval, ECOG PS Eastern Cooperative Oncology Group performance status, HR hazard ratio, TT-B trifluridine/tipiracil plus bevacizumab.

The post-hoc analysis of OS by stratification factors and predefined subgroups showed that treatment effects on OS were either neutral (i.e. favoured neither TT-B nor C-B) or favoured TT-B (Fig. 3). In particular, TT-B had a favourable effect on OS in women (HR 0.44; 95% CI: 0.26–0.76), in those who had not previously undergone surgical resection (HR 0.45; 95% CI: 0.23–0.88), in patients with mutant *BRAF* status (HR 0.17; 95% CI: 0.04–0.68) and in those with an ECOG PS of 2, although the latter did not reach statistical significance (HR 0.50; 95% CI: 0.2–1.26).

### Subsequent treatment

Of those who stopped randomised treatment because of TEAEs, progressive disease or another reason, 77 (50.3%) received at least one subsequent line of treatment (Table 2). More patients received subsequent treatments in the TT-B arm (59.7%) compared with the C-B arm (40.8%). The most commonly used subsequent agents were 5-fluorouracil (30.7%), irinotecan (28.8%) and oxaliplatin (27.5%). Seventeen patients (22.1%) in the TT-B arm subsequently received capecitabine, while only five patients (6.6%) in the C-B arm subsequently received trifluridine/tipiracil.

**Table 2:** Subsequent treatments in patients withdrawing from the TASCO1 trial, in descending order of frequency.

Treatment	TT-B (*n* = 77)		C-B (*n* = 76)		All (*n* = 153)	
	*n*	%	*n*	%	*n*	%
*At least one treatment*	46	59.7	31	40.8	77	50.3
5-Fluorouracil	31	40.3	16	21.1	47	30.7
Irinotecan	28	36.4	16	21.1	44	28.8
Oxaliplatin	25	32.5	17	22.4	42	27.5
Capecitabine	17	22.1	11	14.5	28	18.3
Bevacizumab	8	10.4	8	10.5	16	10.5
Cetuximab	5	6.5	3	3.9	8	5.2
Trifluridine/tipiracil	1	1.3	5	6.6	6	3.9
Panitumumab	4	5.2	2	2.6	6	3.9
Regorafenib	3	3.9	3	3.9	6	3.9
Aflibercept	5	6.5	–	–	5	3.3
Tegafur	2	2.6	2	2.6	4	2.6
Not specified	2	2.6	–	–	2	1.3
Atezolizumab	1	1.3	–	–	1	0.01
Nivolumab	1	1.3	–	–	1	0.01
Ramucirumab	1	1.3	–	–	1	0.01
Mitomycin C	–	–	1	1.3	1	0.01

*C-B* capecitabine plus bevacizumab, *TT-B* trifluridine/tipiracil plus bevacizumab.

### Safety

The safety results were consistent with previously reported data [17]. All patients in the TT-B arm and 74 patients (97.4%) in the C-B arm experienced at least one TEAE. Severe TEAEs (Grade 3 or 4, as defined in the CTCAE [18]) were reported in 68 patients (88.3%) in the TT-B arm and 54 (71.1%) in the C-B arm.

The most common non-haematological TEAEs in patients receiving TT-B were gastrointestinal effects (diarrhoea, nausea, vomiting, decreased appetite and constipation) and fatigue (Table 3). The vast majority of these TEAEs were of mild or moderate intensity. Hypertension occurred in 13 patients (16.9%) and was considered to be severe in most cases. Elevations in alanine aminotransferase (ALT) and aspartate aminotransferase (AST) were detected in nine (11.7%) and eight (10.4%) patients, respectively, in the TT-B arm, but were rated severe in only one patient. Gastrointestinal TEAEs and fatigue were also common among patients receiving C-B, but less so than in the TT-B arm. In the C-B arm, the most frequently reported non-haematological TEAE was hand-foot syndrome (palmar-plantar erythrodysaesthesia), which was reported in 40 patients (52.6%) and was rated as severe in nine (11.8%). This TEAE was uncommon in patients who received TT-B (*n* = 3; 3.9%).

**Table 3:** Frequency of treatment-emergent adverse events (TEAEs) reported in ≥10% of patients in one or both treatment groups in the TASCO1 trial.

	TT-B (*n* = 77)		C-B (*n* = 76)	
	All grades	Grade ≥ 3	All grades	Grade ≥ 3
*Non-haematological events, n (%)*				
Diarrhoea	42 (54.5)	1 (1.3)	34 (44.7)	7 (9.2)
Nausea	38 (49.4)	2 (2.6)	14 (18.4)	–
Fatigue	30 (39.0)	3 (3.9)	23 (30.3)	3 (3.9)
Decreased appetite	29 (37.7)	–	15 (19.7)	1 (1.3)
Vomiting	23 (29.9)	4 (5.2)	10 (13.2)	1 (1.3)
Malignant neoplasm progression	20 (26.0)	13 (16.9)	20 (26.3)	15 (19.7)
Alopecia	18 (23.4)	–	–	–
Stomatitis	14 (18.2)	1 (1.3)	16 (21.1)	–
Asthenia	14 (18.2)	5 (6.5)	18 (23.7)	3 (3.9)
Constipation	13 (16.9)	–	15 (19.7)	–
Weight decreased	13 (16.9)	2 (2.6)	7 (9.2)	–
Hypertension	13 (16.9)	10 (13.0)	9 (11.8)	3 (3.9)
Abdominal pain	11 (14.3)	1 (1.3)	6 (7.9)	1 (1.3)
Urinary tract infection	10 (13.0)	–	5 (6.6)	1 (1.3)
Alanine aminotransferase increased	9 (11.7)	1 (1.3)	2 (2.6)	–
Nasopharyngitis	8 (10.4)	–	5 (6.6)	–
Upper respiratory tract infection	8 (10.4)	1 (1.3)	3 (3.9)	–
Aspartate aminotransferase increased	8 (10.4)	1 (1.3)	3 (3.9)	–
Dizziness	7 (9.1)	–	9 (11.8)	–
Blood bilirubin increased	6 (7.8)	1 (1.3)	11 (14.5)	2 (2.6)
Dyspnoea	6 (7.8)	–	8 (10.5)	1 (1.3)
Cough	3 (3.9)	–	8 (10.5)	–
Palmar-plantar erythrodysaesthesia syndrome	3 (3.9)	–	40 (52.6)	9 (11.8)
*Haematological events*				
Neutropenia	42 (54.5)	36 (46.8)	6 (7.9)	4 (5.3)
Anaemia	26 (33.8)	10 (13.0)	5 (6.6)	–
Neutrophil count decreased	19 (24.7)	15 (19.5)	2 (2.6)	1 (1.3)
White blood cell count decreased	15 (19.5)	9 (11.7)	2 (2.6)	2 (2.6)
Thrombocytopenia	12 (15.6)	3 (3.9)	4 (5.3)	1 (1.3)

Data are presented as the number (%) of patients experiencing each TEAE, up to the cut-off point of 1 September 2020. All TEAEs were graded according to the National Cancer Institute Common Terminology Criteria for Adverse Events version 4.0 [18].

*C-B* capecitabine plus bevacizumab, *TT-B* trifluridine/tipiracil plus bevacizumab.

The proportion of patients who experienced haematological TEAEs was higher in the TT-B arm than in the C-B arm (72.7% and 18.4%, respectively). Grade 3 or 4 neutropenia, anaemia and thrombocytopenia were reported in 46.8%, 13.0% and 3.9% of TT-B recipients, respectively, and in 5.3%, 0.0% and 1.3% of C-B recipients, respectively.

Serious TEAEs were reported in 66.2% and 59.2% of patients in the TT-B and C-B arms, respectively. The percentage of patients who stopped study medication due to a TEAE was 42.9% in the TT-B arm and 42.1% in the C-B arm.

## Discussion

In this report, we present the final OS results of the multicentre TASCO1 study, in which TT-B was investigated as first-line therapy in patients with unresectable mCRC who were considered ineligible for intensive chemotherapy.

Few evidence-based treatment options are available for this patient population. Current National Comprehensive Cancer Network guidelines recommend the use of a fluoropyrimidine, with or without bevacizumab, or for patients with *RAS/BRAF* wild-type tumours and left-sided disease, an anti-EGFR antibody [2]. When designing the TASCO1 study, we selected C-B as the ‘reference’ treatment because of the positive findings of the AVEX trial (see below) [7].

Median OS in TASCO1 was 22.3 months (95% CI: 18.0–23.7) in patients randomised to receive TT-B and 17.7 months (95% CI: 12.6–19.8) in those randomised to receive C-B. We found the effects of randomised treatment on OS to be generally consistent across subgroups of patients defined by age, *RAS* mutation status, ECOG performance status and primary tumour site (left- vs right-sided). In women and patients without prior surgical resection, however, there was a difference between the treatments in favour of TT-B. These findings were consistent with those of the earlier analysis of OS in TASCO1 [17]. The final median OS in the C-B group remains slightly lower than those reported in AVEX (17.7 months versus 21 months), which can be explained by the fact that our study was targeting a different population of patients (higher proportion of ECOG PS2 in TASCO1 15% compared with AVEX 7%).

The results of TASCO1 are notable because of the scarcity of evidence from randomised trials to inform treatment choices in this setting. They are also encouraging, because most patients enrolled in TASCO1 were less fit (i.e. due to older age, worse performance status, comorbidities or other factors), and would therefore be expected to have poorer outcomes than those eligible for intensive treatment. However, median OS times in TASCO1 were not dissimilar to those achieved in earlier phase III randomised trials of first-line FOLFOX or CAPOX with or without bevacizumab (19.6–21.3 months) [19–21]. More recently, median OS times of >25 months have been achieved in trials of FOLFIRI plus bevacizumab and FOLFOXIRI plus bevacizumab [22–25], underlining the need for more effective treatments for patients considered ineligible for these intensive regimens.

TASCO1 is a randomised trial examining the efficacy of first-line treatments for mCRC in a broad spectrum of patients ineligible for oxaliplatin- or irinotecan-containing chemotherapy. The only other controlled trials we identified were three studies investigating fluoropyrimidine-based treatment settings with less stringent criteria for non-eligibility for intensive combination cytotoxic treatment than TASCO1 [7, 11, 26].

In 2005, Kabbinavar and colleagues reported the findings of a randomised (1:1) phase II trial of bevacizumab versus placebo, both in combination with bolus fluorouracil/leucovorin, in 209 patients who were considered unsuitable for first-line irinotecan-containing therapy on the basis of age, performance status or serum albumin level [26]. The addition of bevacizumab to bolus fluorouracil/leucovorin was associated with an increase in median OS of 3.7 months (from 12.9 to 16.6 months), but the difference between treatment groups was not significant (HR 0.79; 95% CI: 0.56–1.10). Similar results were obtained in the AVEX (AVastin in the Elderly with Xeloda) trial, which compared C-B versus capecitabine alone as first-line treatment in patients aged ≥70 years; combination treatment increased median OS by 3.9 months (from 16.8 months to 20.7 months), but again, the difference between treatments was not statistically significant (HR 0.79; 95% CI: 0.57–1.09) [7]. More recently, the novel fluoropyrimidine S-1 was compared with capecitabine (both with or without bevacizumab) in 161 previously untreated patients with mCRC in whom the planned treatment was fluoropyrimidine monochemotherapy (with or without bevacizumab) [11]. Although the objective of the trial was to compare the safety (specifically, the rates of palmar-plantar erythrodysaesthesia) rather than the efficacy of the two treatments, median OS was reported as 17.0 months in the S-1 arm and 17.1 months in the capecitabine arm [27].

The fact that these very similar survival results were obtained in separate clinical trials published over a period of 14 years illustrates the general lack of progress in this therapeutic area, and underscores the need for treatment options that improve survival. The phase II TASCO1 trial was designed to investigate the potential of TT-B as first-line treatment in patients with mCRC for whom treatment options are limited. In the time since TASCO1 was initiated, clinical trials have confirmed that TT-B has the potential to improve outcomes in patients with treatment-refractory mCRC [15, 28]. Phase III comparative trials of TT-B are now underway in both the first-line (SOLSTICE; NCT03869892; vs C-B) and treatment-refractory (SUNLIGHT; NCT04737187) settings [16, 29]. In terms of trial populations, interventions and endpoints, the SOLSTICE trial is very similar to TASCO1. However, the planned sample size of 854 patients in SOLSTICE will allow for statistical comparisons to be made between TT-B and C-B on the primary endpoint of PFS [16]. Additionally, stratification in SOLSTICE is by tumour localisation (right vs left) and reason for ineligibility for intensive therapy (clinical vs non-clinical), as well as by ECOG performance status (0 vs 1 vs 2); in contrast to TASCO1, patients are not stratified by *RAS* mutation status or country. OS is a key secondary endpoint, and the trial will also provide data on response rates, quality of life, safety and tolerability.

We have also presented an updated analysis of TEAEs reported during the TASCO1 trial. These safety results are consistent with those previously reported [17], and there were no new safety signals or unexpected findings. Gastrointestinal adverse effects, such as diarrhoea, occurred commonly with both treatments and were mostly mild or moderate in intensity. As expected, TT-B was associated with a higher incidence of haematological abnormalities than C-B, whereas C-B was frequently associated with palmar-plantar erythrodysaesthesia.

Our trial has several limitations, as previously described [17]. The lack of standardised criteria that clearly define eligibility for intensive treatment may have led to inconsistencies between study centres in patient enrolment. Additionally, patients were not stratified by reason for ineligibility (e.g. age, ECOG performance status or comorbidities); although we included this variable in the subgroup analysis, the results must be interpreted with caution due to the small numbers of patients in some subgroups.

## Conclusions

This final OS analysis of TASCO1 demonstrates that combination treatment with TT-B is efficacious, and has an acceptable safety and tolerability profile, when used as first-line treatment in patients with unresectable mCRC who are ineligible for intensive chemotherapy. TT-B is now being compared with C-B in a much larger clinical trial (SOLSTICE) in this patient population, the results of which are awaited with interest.

## Supplementary information

AJ CHECKLIST

CONSORT checklist

## Data Availability

The data that support the findings of this study are available from Institut de Recherches Internationales Servier, but restrictions apply to the availability of these data, which were used under license for the current study and so are not publicly available. Data are, however, available from the authors upon reasonable request and the study protocol is available at https://clinicaltrials.gov/ct2/show/NCT02743221 and https://www.clinicaltrialsregister.eu/ctr-search/trial/2015-004544-18/GB.

## References

[1] Van Cutsem E, Cervantes A, Adam R, Sobrero A, Van Krieken JH, Aderka D (2016). ESMO consensus guidelines for the management of patients with metastatic colorectal cancer. Ann Oncol..

[2] National Comprehensive Cancer Network. NCCN clinical practice guidelines in oncology (NCCN Guidelines®). Colon cancer. Version 2.2021. 2021. https://www.nccn.org/professionals/physician_gls/default.aspx. Accessed 31 January 2021.

[3] Cremolini C, Antoniotti C, Stein A, Bendell J, Gruenberger T, Rossini D (2020). Individual patient data meta-analysis of FOLFOXIRI plus bevacizumab versus doublets plus bevacizumab as initial therapy of unresectable metastatic colorectal cancer. J Clin Oncol.

[4] Cremolini C (2019). When to use triplet chemotherapy as first-line treatment in metastatic colorectal cancer. Clin Adv Hematol Oncol..

[5] Bossé D, Vickers M, Lemay F, Beaudoin A (2015). Palliative chemotherapy for patients 70 years of age and older with metastatic colorectal cancer: a single-centre experience. Curr Oncol..

[6] Bruera G, Russo A, Galvano A, Rizzo S, Ricevuto E (2017). Clinical parameters to guide decision-making in elderly metastatic colorectal cancer patients treated with intensive cytotoxic and anti-angiogenic therapy. Oncotarget.

[7] Cunningham D, Lang I, Marcuello E, Lorusso V, Ocvirk J, Shin DB (2013). Bevacizumab plus capecitabine versus capecitabine alone in elderly patients with previously untreated metastatic colorectal cancer (AVEX): an open-label, randomised phase 3 trial. Lancet Oncol..

[8] Dagher M, Sabido M, Zollner Y (2019). Effect of age on the effectiveness of the first-line standard of care treatment in patients with metastatic colorectal cancer: systematic review of observational studies. J Cancer Res Clin Oncol.

[9] Pinto C, Antonuzzo L, Porcu L, Aprile G, Maiello E, Masi G (2017). Efficacy and safety of bevacizumab combined with fluoropyrimidine monotherapy for unfit or older patients with metastatic colorectal cancer: a systematic review and meta-analysis. Clin Colorectal Cancer.

[10] Tapia Rico G, Price T, Tebbutt N, Hardingham J, Lee C, Buizen L (2019). Right or left primary site of colorectal cancer: outcomes from the molecular analysis of the AGITG MAX trial. Clin Colorectal Cancer.

[11] Kwakman JJM, Simkens LHJ, van Rooijen JM, van de Wouw AJ, Ten Tije AJ, Creemers GJM (2017). Randomized phase III trial of S-1 versus capecitabine in the first-line treatment of metastatic colorectal cancer: SALTO study by the Dutch Colorectal Cancer Group. Ann Oncol..

[12] European Medicines Agency. Lonsurf Summary of Product Characteristics. 2016. https://www.ema.europa.eu/en/documents/product-information/lonsurf-epar-product-information_en.pdf. Accessed 31 January 2021.

[13] Lenz HJ, Stintzing S, Loupakis F (2015). TAS-102, a novel antitumor agent: a review of the mechanism of action. Cancer Treat Rev.

[14] Tsukihara H, Nakagawa F, Sakamoto K, Ishida K, Tanaka N, Okabe H (2015). Efficacy of combination chemotherapy using a novel oral chemotherapeutic agent, TAS-102, together with bevacizumab, cetuximab, or panitumumab on human colorectal cancer xenografts. Oncol Rep..

[15] Kuboki Y, Nishina T, Shinozaki E, Yamazaki K, Shitara K, Okamoto W (2017). TAS-102 plus bevacizumab for patients with metastatic colorectal cancer refractory to standard therapies (C-TASK FORCE): an investigator-initiated, open-label, single-arm, multicentre, phase 1/2 study. Lancet Oncol..

[16] André T, Saunders M, Kanehisa A, Gandossi E, Fougeray R, Amellal NC (2020). First-line trifluridine/tipiracil plus bevacizumab for unresectable metastatic colorectal cancer: SOLSTICE study design. Future Oncol..

[17] Van Cutsem E, Danielewicz I, Saunders MP, Pfeiffer P, Argiles G, Borg C (2020). Trifluridine/tipiracil plus bevacizumab in patients with untreated metastatic colorectal cancer ineligible for intensive therapy: the randomized TASCO1 study. Ann Oncol..

[18] U.S. Deparment of Health and Human Services. Common Terminology Criteria for Adverse Events (CTCAE). Version 4.03. 2010. https://evs.nci.nih.gov/ftp1/CTCAE/CTCAE_4.03/CTCAE_4.03_2010-06-14_QuickReference_8.5x11.pdf. Accessed 31 March 2021.

[19] Saltz LB, Clarke S, Díaz-Rubio E, Scheithauer W, Figer A, Wong R (2008). Bevacizumab in combination with oxaliplatin-based chemotherapy as first-line therapy in metastatic colorectal cancer: a randomized phase III study. J Clin Oncol..

[20] Cassidy J, Clarke S, Díaz-Rubio E, Scheithauer W, Figer A, Wong R (2008). Randomized phase III study of capecitabine plus oxaliplatin compared with fluorouracil/folinic acid plus oxaliplatin as first-line therapy for metastatic colorectal cancer. J Clin Oncol..

[21] Ducreux M, Bennouna J, Hebbar M, Ychou M, Lledo G, Conroy T (2011). Capecitabine plus oxaliplatin (XELOX) versus 5-fluorouracil/leucovorin plus oxaliplatin (FOLFOX-6) as first-line treatment for metastatic colorectal cancer. Int J Cancer.

[22] Fuchs CS, Marshall J, Barrueco J (2008). Randomized, controlled trial of irinotecan plus infusional, bolus, or oral fluoropyrimidines in first-line treatment of metastatic colorectal cancer: updated results from the BICC-C study. J Clin Oncol.

[23] Heinemann V, von Weikersthal LF, Decker T, Kiani A, Kaiser F, Al-Batran SE (2021). FOLFIRI plus cetuximab or bevacizumab for advanced colorectal cancer: final survival and per-protocol analysis of FIRE-3, a randomised clinical trial. Br J Cancer.

[24] Cremolini C, Loupakis F, Antoniotti C, Lupi C, Sensi E, Lonardi S (2015). FOLFOXIRI plus bevacizumab versus FOLFIRI plus bevacizumab as first-line treatment of patients with metastatic colorectal cancer: updated overall survival and molecular subgroup analyses of the open-label, phase 3 TRIBE study. Lancet Oncol.

[25] Heinemann V, von Weikersthal LF, Decker T, Kiani A, Vehling-Kaiser U, Al-Batran SE (2014). FOLFIRI plus cetuximab versus FOLFIRI plus bevacizumab as first-line treatment for patients with metastatic colorectal cancer (FIRE-3): a randomised, open-label, phase 3 trial. Lancet Oncol.

[26] Kabbinavar FF, Schulz J, McCleod M, Patel T, Hamm JT, Hecht JR (2005). Addition of bevacizumab to bolus fluorouracil and leucovorin in first-line metastatic colorectal cancer: results of a randomized phase II trial. J Clin Oncol..

[27] Kwakman JJM, van Werkhoven E, Simkens LHJ, van Rooijen JM, van de Wouw YAJ, Tije AJT (2019). Updated survival analysis of the randomized phase III trial of S-1 versus capecitabine in the first-line treatment of metastatic colorectal cancer by the Dutch Colorectal Cancer Group. Clin Colorectal Cancer.

[28] Pfeiffer P, Yilmaz M, Möller S, Zitnjak D, Krogh M, Petersen LN (2020). TAS-102 with or without bevacizumab in patients with chemorefractory metastatic colorectal cancer: an investigator-initiated, open-label, randomised, phase 2 trial. Lancet Oncol.

[29] Tabernero J, Taieb J, Prager GW, Ciardiello F, Fakih M, Leger C (2021). Trifluridine/tipiracil plus bevacizumab for third-line management of metastatic colorectal cancer: SUNLIGHT study design. Future Oncol..

